# Long-Latency Feedback Coordinates Upper-Limb and Hand Muscles during Object Manipulation Tasks^1^,^2^,^3^

**DOI:** 10.1523/ENEURO.0129-15.2016

**Published:** 2016-03-10

**Authors:** Frédéric Crevecoeur, Jean-Louis Thonnard, Philippe Lefèvre, Stephen H. Scott

**Affiliations:** 1 Institute of Communication Technologies, Electronics and Applied Mathematics (ICTEAM), Université catholique de Louvain, Louvain-la-Neuve 1348, Belgium; 2 Institute of Neuroscience (IoNS), Université catholique de Louvain, Louvain-la-Neuve 1348, Belgium; 3 Physical and Rehabilitation Medicine Department, Cliniques Universitaires Saint-Luc, Louvain-la-Neuve 1348, Belgium; 4 Centre for Neuroscience Studies, Queen’s University, Kingston, Ontario K7L3N6, Canada; 5 Department of Biomedical and Molecular Science, Queen’s University, Kingston, Ontario K7L3N6, Canada

**Keywords:** feedback control, grip force control, motor prediction, object manipulation

## Abstract

Suppose that someone bumps into your arm at a party while you are holding a glass of wine. Motion of the disturbed arm will engage rapid and goal-directed feedback responses in the upper-limb. Although such responses can rapidly counter the perturbation, it is also clearly desirable not to destabilize your grasp and/or spill the wine. Here we investigated how healthy humans maintain a stable grasp following perturbations by using a paradigm that requires spatial tuning of the motor response dependent on the location of a virtual target. Our results highlight a synchronized expression of target-directed feedback in shoulder and hand muscles occurring at ∼60 ms. Considering that conduction delays are longer for the more distal hand muscles, these results suggest that target-directed responses in hand muscles were initiated before those for the shoulder muscles. These results show that long-latency feedback can coordinate upper limb and hand muscles during object manipulation tasks.

## Abstract (precis)

Suppose that someone bumps into your arm at a party while you are holding a glass of wine. Motion of the disturbed arm will engage rapid and goal-directed feedback responses in the upper-limb.

## Visual Overview

**Figure:**
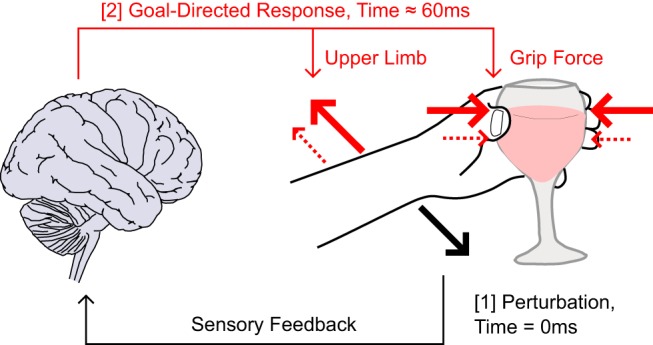

## Significance Statement

Skilled object manipulation relies on fine control of finger forces applied on the held objects. A prevailing hypothesis suggests that the nervous system predicts the consequence of motor commands to anticipate self-generated loads arising when we move the objects around. Here we show that following an external perturbation, motor responses in upper-limb and hand muscles expressed synchronized, target-directed modulation in ∼60 ms. This finding cannot be explained by internal predictions from forward models, as processing and conduction times expected in this framework imply measurable delays between the expression of flexible feedback in upper-limb and hand muscles. Instead, our results suggest that in such context, stable control of grasp is also mediated by goal-directed feedback coordination of upper-limb and hand muscles.

## Introduction

Humans and other primates have evolved complex neural functions subserving skilled manipulation of objects and tools (Johansson and Flanagan, 2009). A well -documented aspect of motor control during object manipulation is that the grip force applied on held objects is modulated with the loads arising when we move the object around (Westling and Johansson, 1984; Flanagan and Wing, 1997; Danion and Sarlegna, 2007; Diamond et al., 2015; Hadjiosif and Smith, 2015). This modulation of grip force during self-generated movements occurs in phase with changes in load force, suggesting that neural control of grasp relies on internal predictions (Wolpert and Flanagan, 2001). Formally, current theories suggest that forward models predict the sensory consequences of motor commands based on an efferent copy, allowing anticipatory grip force adjustments ahead of sensory feedback (Kawato, 1999; Wolpert and Flanagan, 2001; Shadmehr et al., 2010; Wolpert et al., 2011).

In the context of feedback response to external disturbances, as when someone bumps into your arm while you are holding a glass of wine, the consequences of motor commands cannot be anticipated so easily. The difficulty in this context arises from the fact that mechanical perturbations evoke a stretch response initially generated in the motor periphery and spinal cord (in ∼20 ms for upper-limb muscles), followed by flexible and goal-directed feedback in ∼50 ms (long-latency; Scott, 2012). Thus, following a perturbation, forward models in the CNS can only start influencing grip force control after upper-limb feedback commands are already engaged. Considering the flexibility of rapid motor responses to mechanical perturbations, the question whether and how the nervous system maintains a stable grip following perturbations of the upper limb remains open.

Several mechanisms may contribute to maintaining a stable grip in such situation. One simple candidate mechanism is a default increase in grip force generated by the occurrence of the perturbation, and aimed to counter any load constraint independent of the upper limb motor correction. Another mechanism is the internal prediction of the consequences of upper-limb motor commands mediated by forward models as suggested in the context of voluntary movements. In principle, this mechanism enables more flexibility in the grip control but at the cost of delaying the grip force adjustments, which is potentially detrimental given that upper-limb feedback responses can exhibit important modulation in ∼50 ms. A third hypothesis is the coupling of upper-limb and grip-force control in the feedback control law allowing synchronized, task-specific motor responses.

Here we examine this issue by using a paradigm known to elicit robust modulation of motor responses dependent on whether the perturbation pushes the limb toward or away from the goal target (Pruszynski et al., 2008). We leverage this paradigm in the context of object manipulation to measure whether and when grip force reflects knowledge of upper-limb motor commands. The timing of target-dependent modulation in grip control provides important insight on the underlying mechanism, because both internal processing (eg, ∼10 ms for internal cortico-cerebellar feedback; Allen and Tsukahara, 1974), and additional transmission delays to the more distal musculature for grasp control (∼10 ms; Ingram et al., 1987; Wang et al., 1999; Devanne et al., 2006) impose physiological constraints on grip-force modulation mediated by forward models. In other words, if the nervous system performs predictions in a serial way following a perturbation, we should observe measurable delays between the onset of task specific feedback in upper limb and hand muscles. Contrasting with this prediction, our data highlight that the onset of target-dependent modulation occurred at ∼60 ms in both upper-limb and hand muscles. These results emphasize that the coupling between finger forces and inertial loads when countering external disturbances may result from direct feedback coordination, which synchronizes upper-limb and hand motor systems according to task demands.

## Materials and Methods

Sixteen healthy participants (10 females) between 18 and 42 years of age took part in the study after providing written informed consent following standard procedures approved by the ethics committee at Queen’s University.

### Experimental procedures

Participants were seated comfortably in front of a virtual reality display projecting visual targets and a hand-aligned cursor. The participants’ right arm was placed in a KINARM exoskeleton providing support against gravity and allowing motion in the horizontal plane (Scott, 1999; KINARM, BKIN Technologies). All participants performed the three tasks presented below. The order in which the distinct tasks were performed was counterbalanced across participants.

#### Task 1: goal-directed shoulder response

**Figure 1.**
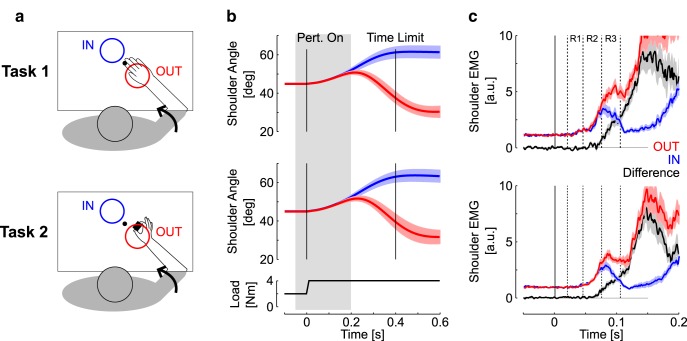
***a***, Illustration of the experimental conditions. Participants were instructed to reach for the goal target as soon as they felt the perturbation. Perturbation-evoked motion was directed either toward (IN, blue) or away from (OUT, red) the goal target. Only one target was displayed at the beginning of each trial. The two targets are shown for illustrative purposes. The task was performed with the hand strapped on a horizontal support (Task 1, top) or with an instrumented object held in precision grip (Task 2, bottom). In this condition, the hand and object were not supported against gravity. A flexor background load of 2 Nm was applied to pre-activate the shoulder extensor (posterior deltoid) and the analysis focuses on flexion perturbations. ***b***, Average shoulder displacement ±SEM across participants in Task 1 (top) and Task 2 (middle). The applied load is illustrated in the bottom plot. The shaded area represents the time window expanded in ***c***. Vertical lines illustrate the perturbation onset and time limit imposed to reach for the target (400 ms). ***c***, Perturbation-evoked response of the shoulder extensor muscle (mean ± SEM across participants) with color code as in ***a*** and ***b***. The black traces illustrate the paired difference in response across IN and OUT perturbation trials. Pre-perturbation (Pre) was from −50 to 0 ms and the early voluntary epoch (Vol) was from 120 to 180 ms. Vertical dashed lines delineate the different epochs of motor responses.

The goal of this task was to replicate previously published results on directional tuning of rapid motor responses to perturbation applied to the upper limb (Pruszynski et al., 2008). Participants were instructed to stabilize their fingertip in the center target (radius 0.6 cm) corresponding to 45° and 90° of shoulder and elbow angles, respectively. A constant flexor load of +2 Nm was applied on the shoulder to pre-activate the shoulder extensor (posterior deltoid). A large goal target was initially displayed as an open circle in one of two possible locations [center (−6, 6) cm or (6, −6) cm relative to the fingertip coordinates in the initial joint configuration, radius 7.5 cm; Fig. 1*a*]. Flexion or extension perturbations were applied in addition to the background load (±2 Nm, 10 ms buildup) following a random delay uniformly distributed between 2 and 4 s after stabilization in the center target. The goal target simultaneously filled in. Participants were instructed to reach for the goal target as soon as they felt the perturbation. The goal-target turned green to indicate that the target was acquired within the prescribed time limit (<400 ms), or red otherwise. The location of the goal target and the perturbation direction were randomized to avoid anticipation. Participants performed two blocks of 40 trials, 10 per combination of target location and perturbation direction.

We focused our analyses on the shoulder flexion perturbations evoking a stretch response in the posterior deltoid (+2 Nm), as it provides a robust measurement of the moment when target-directed response component inhibits or enhances the initial default stretch response. Overall, the extension perturbation, unloading the shoulder, evoked later target-directed modulation in both upper limb and hand muscles. Targets can be labeled as “IN” or “OUT” dependent on whether the perturbation pushed the hand toward the farther target (Fig. 1*a*, IN) or away from the nearer target (Fig. 1*a*, OUT).

#### Task 2: goal-directed shoulder and grip response with a held load

Building on the results of Task 1, Task 2 was designed to extract the moment when changes in grip force reflect knowledge of the upper limb feedback correction. The task was similar to Task 1, except that participants were instructed to hold an instrumented object between their thumb and index finger (Fig. 1*a*, Task 2). The object was composed of a one-dimensional strain gauge measuring compression and mounted with aluminum cylinders on each side for finger placement (WMC-250 load cell interface). The grip aperture was 3.5 cm and the total mass was 330g. Participants’ hand and the held object were not supported against gravity. Participants were allowed to interrupt the ongoing block of trials at any time in case they experienced fatigue. In most cases, the few minutes of rest given between blocks were sufficient. As a result, no participant dropped the object during the experiment. The center and goal targets were displayed at the same locations as Task 1 (Fig. 1a). Participants performed three blocks of 40 trials (10 trials per target/perturbation combination).

#### Task 3: visually cued reaching with a held load

We used this task to measure voluntary reaction times to address the possibility that rapid visuomotor feedback contributed to the motor response found in Task 2. Participants were instructed to stabilize in the start target against the background load, and to reach for the goal target as soon as it filled in. Participants were holding the instrumented object in precision grip as in Task 2. There was no perturbation applied on the shoulder. The goal target was initially displayed as an open circle and the cue to reach was delivered only if the cursor remained stable in the start target for a time period uniformly distributed between 2 and 4 s. This procedure allowed us to avoid false starts while minimizing the uncertainty about the movement to execute. Participants performed one block of 40 trials (20 trials per target location).

### Data collection and analysis

All signals were sampled at 1 kHz and digitally filtered with zero-lag, fourth-order Butterworth filters with cutoff frequencies as specified below. Shoulder and elbow angles were low-pass filtered with cutoff frequency set to 20 Hz. The shoulder and elbow angles were used to compute the linear acceleration of the held load. The component of the acceleration normal to the grip axis was then multiplied by the object mass to derive an estimate of the inertial loads acting tangentially at the interface between the fingertips and the held object. For Tasks 2 and 3, grip force signals were low-pass filtered with a 20 Hz cutoff frequency. We collected the activity of the shoulder extensor muscle (posterior deltoid), as well as hand muscles involved in grip force generation. The electrodes were placed on the first dorsal interosseous and on the contractile part of the thenar eminence following palpation during pinch-force generation. Activity from the electrode placed on the thenar eminence reflected a combination of several muscles, of which at least two muscles correlate well with grip-force production (flexor pollicis brevis and adductor pollicis; Maier and Hepp-Reymond, 1995). As a consequence, signals from the two electrodes attached on the hand were averaged and are designated as “hand muscles” because of the difficulty to isolate individual muscles with surface electrodes. Muscle activity was digitally band-pass filtered (10–500 Hz) and averaged across trials for each task. The activity of the posterior deltoid was normalized to the grand-average activity evoked by the background load across all trials. The normalization of hand muscles was performed for each trial relative to the average activity measured prior to the perturbation. We used this procedure because, unlike the shoulder muscle, there was no reference grip force imposed prior to the perturbation that could be used for normalization. Thus, changes in EMG of hand muscles are measured relative to the baseline activity of each individual trial. Binned analysis of muscle activity was based on average muscle responses across epochs following standard definition (Pruszynski et al., 2008): pre-perturbation (−50 to 0 ms), R1 (20–45 ms), R2 (45–75 ms), R3 (75–105 ms), and early voluntary (Vol; 120–180 ms). All epochs are defined relative to the shoulder perturbation onset. Comparisons of EMG activity across conditions and epochs were performed with paired *t* tests. Linear regressions were performed to address the relationship between muscle responses across muscle samples and between motor responses and grip force generation. Regressions were first performed on individual trials for each participant independently to avoid that idiosyncratic differences impacted the results. We also validated the results of the independent linear regressions by computing mixed linear models treating participants as a random factor.

The average response across predefined epochs was used to highlight the presence of significant differences following standard definitions. We then addressed the moment when muscle activity collected following OUT trials started diverging from that of IN trials as accurately as possible to set physiological constraints on the underlying mechanism. To do so, we computed an estimate of response overlap across IN and OUT trials for each time step with receiver operating characteristic curves (ROCs; Metz, 1978). We adopted the convention that two overlapping distributions corresponded to ROC=0.5, and strictly nonoverlapping distributions corresponded to ROC=1. Time series of ROC from all participants were compared with chance levels using a sliding *t* test based on a centered 20 ms moving average (from −10 to +10 ms relative to each point in time). The moment when the sliding *p* value dropped <0.05 was used to determine the onset of response divergence across IN and OUT perturbation trials. We used the moment when the time series of *p* values become significant instead of estimating the onset of divergence to mitigate the influence of the 20 ms sliding window. This procedure allowed us to quantify the trial-by-trial overlap for each participant, and then perform a statistical test on the moment when the overlap across participants becomes significantly different from chance.

This approach was developed because EMG measurements were too variable to estimate the onset of the target-directed response for each participant separately based on ROCs or on sliding (unpaired) *t* tests from individual trials. Thus, it was necessary to perform statistical comparisons of ROCs from all participants pooled together with the level of 0.5 corresponding to perfectly overlapping distributions. We verified the extent to which the sliding window of 20 ms on time series of ROCs affected the estimate of the response onset by generating artificial series composed of a linear increase starting at known time step, plus a white noise with variance equal to the variance of ROCs from one participant picked at random with replacement. The slope of the linear trend was set to the slope of the average ROCs of hand or shoulder muscles. Sixteen series were generated to match our empirical sample size. This procedure was repeated 1000 times to generate confidence intervals on the estimate of the response latency. When considering the slope of the average ROC from hand muscles, the difference between the estimated and true onset time was 5±4 ms. Thus, the estimates were slightly pushed toward later values, but not significantly different from the true value. When testing the method with a slope corresponding to the average of shoulder ROCs, the estimate was −1±3 ms. Performing the same test without sliding window, or with a window of smaller width, increased the average error. In all, the sliding window partially mitigates the impact of noise, and our method provides reliable estimates of onset times.

Finally, we used a variant of the Jackknife estimation of variance for the goal-directed response latency (Efron and Stein, 1981). We computed the latencies in shoulder and hand muscles after leaving out 10% of trials randomly selected from each individual dataset. We performed identical measurements of time-varying ROCs across the remaining trials, followed by the sliding *t* test. This procedure was repeated 200 times on data from Task 2.

## Results

Participants interacted with a robotic device equipped with a virtual reality display projecting the visual targets. Following stabilization of the hand aligned cursor in the start target, participants were instructed to reach and stabilize in the goal target as soon as they felt the perturbation. Importantly, the perturbation pushed their limb either toward (Fig. 1*a*, IN) or away (Fig. 1*b*, OUT) from the goal target (see Materials and Methods). Thus, succeeding at the task required spatial tuning of upper-limb motor responses according to the location of the goal target (Pruszynski et al., 2008). Participants performed the task with their hand strapped on a horizontal support (Fig. 1*a*, Task 1), or with an instrumented object held in precision grip (Fig. 1*a*, Task 2). Clearly, maintaining a stable grip during Task 2 requires that the grip force applied on the object be sufficient to overcome the inertial loads resulting from upper-limb feedback corrections. Participants also performed visually cued reaching with the object held in precision grip to measure response times associated with visually cued reaching (Task 3; see Materials and Methods).

Shoulder traces following the perturbations are represented in Figure 1*b*. Observe the rapid divergence in shoulder trajectories dependent on the location of the goal target in both Tasks 1 and 2. Task 1 reproduced the rapid differentiation of muscle response according to the location of the goal target (Pruszynski et al., 2008). The perturbation first evoked a significant short-latency response (Fig. 1*c*; R1>Pre; mean EMG across epochs: *t*_(15)_>4.28, *p*<0.001), which was undifferentiated across IN and OUT perturbations, as a consequence of the similar stretch that followed the perturbation (R1_IN_≈R1_OUT_; *t*_(15)_=0.92, *p*=0.37). Shoulder activity started to reflect target-directed responses within the R2 epoch (R2_OUT_>R2_IN_; *t*_(15)_=3.41, *p*<0.01), and the contrast between IN and OUT responses further increased in the subsequent epochs (R3_OUT_>R3_IN_ and Vol_OUT_>Vol_IN_; *t*_(15)_>7, *p*<10^−5^).

In Task 2, we measured an overall decrease in activity for all epochs (*t*_(15)_>3.5, *p*<0.001), suggesting a default downregulation of upper-limb muscle activity when the task involved the manipulation of an object. The baseline activity in Task 1 was on average 32% greater than the baseline activity in Task 2. Besides this default decrease, we observed a rapid differentiation of feedback response qualitatively similar to Task 1, except that the contrast between IN and OUT trials within the R2 was only marginally significant (Fig. 1*c*, bottom; R2_OUT_>R2_IN_; *t*_(15)_=1.62, *p*=0.062; R3_OUT_>R3_IN_ and Vol_OUT_>Vol_IN_; for both tests: *t*_(15)_>7, *p*<0.001).

**Figure 2.**
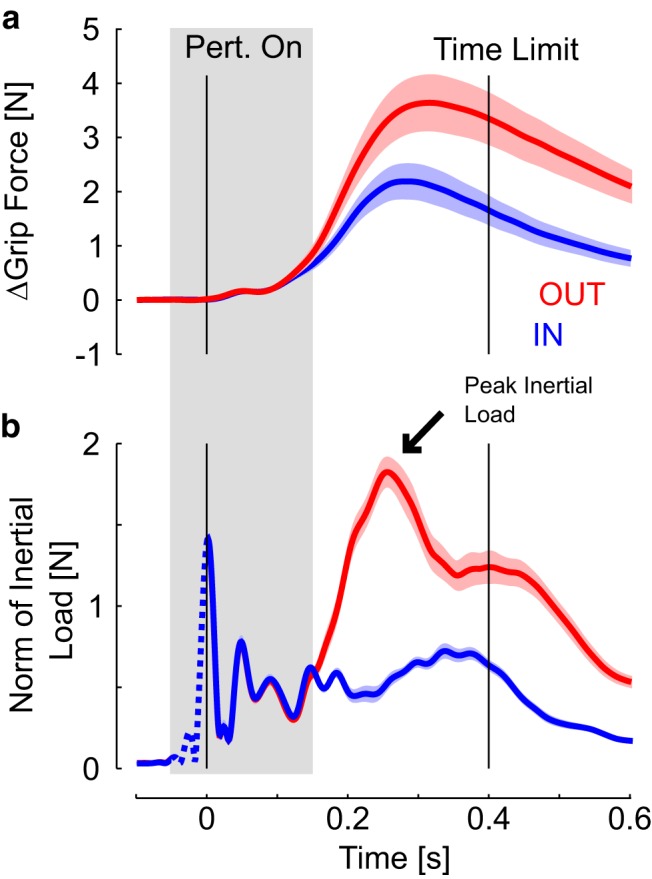
*a*, Average grip force response for IN and OUT trials depicted in blue and red, respectively. Colored areas represent 1 SEM across individuals. Perturbation onset and maximum time given to reach for the goal target are represented as in Figure 1*b*. *b*, Inertial load computed as the object linear acceleration multiplied by its mass with identical color code as in ***a***. Dashed portion of the load profiles are the consequence of the digital filter. The gray shaded area represents the time window expanded in Figure 3.

Inertial loads and grip feedback responses to the mechanical perturbations are illustrated in Figure 2 with identical color code as in Figure 1. Figure 2 illustrates that the feedback responses following OUT trials generated a peak in the load profile arising shortly after the perturbation (∼260 ms on average), requiring higher levels of grip force to maintain the object stable in comparison with IN trials (Fig. 2*b*, black arrow). Accordingly, grip force displayed a clear modulation across IN and OUT perturbations. It is also visible in Figure 2 that the inertial loads (and end-point acceleration) were similar across IN and OUT trials for the first ∼100 ms. Compatible with the similar shoulder displacements shown in Figure 1*b*, the similarity in inertial loads is another verification that sensory feedback about the limb motion or from the finger–object interface was similar across IN and OUT perturbations at the moment when the muscle response from the shoulder started reflecting target-directed feedback.

**Figure 3.**
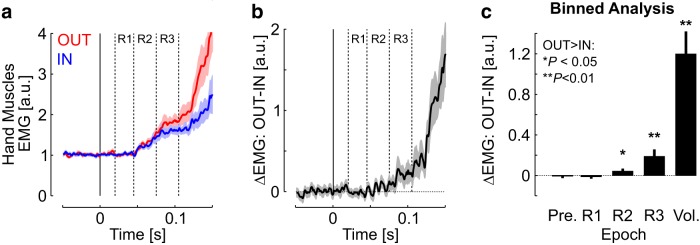
***a***, Perturbation-evoked response in hand muscles averaged across muscles and participants. The dashed lines illustrate the short-latency (R1, 20–45 ms) and long-latency (R2, 45–75 ms; R3, 75–105 ms) epochs following standard definitions (see Materials and Methods). ***b***, Difference in hand EMG between OUT and IN perturbations. ***c***, Average difference between OUT and IN perturbations-related EMG responses across the different epochs. Significant differences at the level *p*<0.05 (*p*<0.01) are reported with one (two) star(s).

Examination of the force response immediately after the perturbation revealed the first increase in grip force occurring almost immediately after the perturbation (Fig. 2*a*; <40 ms), which is too quick to result from changes in muscles activity (Fig. 3*a*). This increase likely reflected mechanical interactions, such as small changes in grip configuration or the Poynting effect, characterized by a force normal to the direction of a shear stress applied to tissue with nonlinear elasticity, such as the skin (Misra et al., 2010). A closer look at the normal force revealed that, on average, the effect of this mechanical interaction started at ∼15 ms following the onset of the commanded torque applied to the shoulder. The perturbation did not evoke any significant response in the recorded hand muscles during the R1 epoch (Fig. 3*a*; Pre≈R1; *t*_(15)_<1, *p*>0.2). A significant EMG response was observed in the R2 epoch (R2>Pre; *t*_(15)_>5, *p*<0.001), leading to further increase in grip force. Strikingly, evidence for target-dependent responses in hand muscles emerged during the R2 epoch (Fig. 3), as revealed by significant differences across IN and OUT perturbation responses (Fig. 3; R2_OUT_>R2_IN_; one-tail comparison, *t*_(15)_>1.8, *p*<0.05). The contrast between IN and OUT perturbations increased across the following epochs (R3_OUT_>R3_IN_; *t*_(15)_>2.91, *p*<0.05; Vol_OUT_>Vol_IN_; *t*_(15)_>5.5, *p*<0.001). Recall that all response epochs are defined relative to the shoulder perturbation onset.

**Figure 4.**
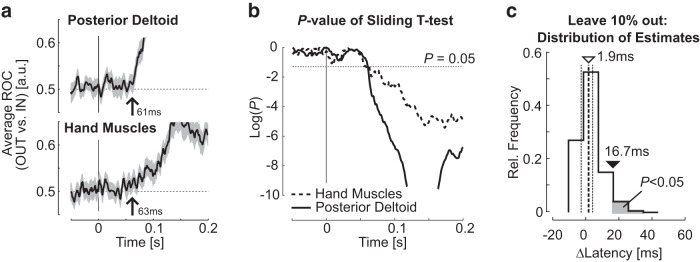
***a***, Ensemble average of ROC across participants (mean ± SEM) as a function of time for shoulder (top) and hand (bottom) muscles. Traces were smoothed with a 5 ms sliding window for illustration purposes. Vertical arrows indicate the moment when the sliding *t* test becomes significant. ***b***, *p* values of the sliding *t* test comparing participants ROCs to chance level as a function of time for hand (dashed) and shoulder (solid) muscles. ***c***, Distribution of difference between the latency of goal-directed feedback response in hand and shoulder muscles obtained with 200 repeats of leave-10%-out Jackknife procedure (ΔLatency > 0 means that the shoulder muscle leads hand muscles). Vertical dashed lines represent the median (thick line: 1.9 ms, open triangle) and the interquartile range [thin lines, (−2.5, 4.3) ms]. The 95th percentile of the distribution is represented with the filled triangle. The gray area represents the results less likely than 0.05.

Thus, EMG signals in shoulder and hand muscles started reflecting target-directed feedback in the same epoch (R2). We measured the onset of this target-directed response component as accurately as possible based on the ROC analyses explained above. For these analyses only, EMG from the posterior deltoid was normalized to the baseline activity prior to the perturbation for each trial independently, as for hand muscles. Time series of ROCs are illustrated in Figure 4, *a* and *b*, with the measurement of the onset of target-directed modulation (mean ± SE across participants). Recall that this time represents the moment when the trial-by-trial overlap between IN and OUT response distributions across participants becomes significantly different from the value of 0.5, corresponding to chance level. Figure 4*c* shows the difference between goal-dependent response latency obtained by randomly leaving out 10% of trials and reproducing the same measurements for shoulder and hand muscles. The interquartile range was −2.5 to 4.3 ms (Fig. 4*c*, dotted vertical lines), and the 95th percentile was 16.7 ms (Fig. 4*c*, black triangle).

Figure 4*a* shows that for the shoulder muscle presenting with very abrupt changes in ROC, the possible bias induced by the moving average is rather small and the extracted timing appears reliable. Lowering the threshold shown in Figure 4*b* should in principle yield more conservative estimates, but doing so does not necessarily improve accuracy. For instance, the time series of *p* values for hand muscles crosses the threshold of *p*=0.01 at ∼80 ms. However, the average activity in R2 already exhibited significant differences across IN and OUT perturbations (Fig. 3). Thus, a more conservative threshold provides estimates that are biased toward later values, which is also problematic. Although the exact timing may be difficult to measure based on EMG, our data clearly show that the response differentiation started in R2. In addition, the time varying profile of *p* values presented similar trend for shoulder and hand muscles, with a clear inflexion starting at ∼50 ms. Notably, because our method overestimated the true latency for shallower slopes (see Materials and Methods), we believe that our estimate of the difference between the onset of goal-directed response in upper limb and hand muscles is in fact conservative.

**Figure 5.**
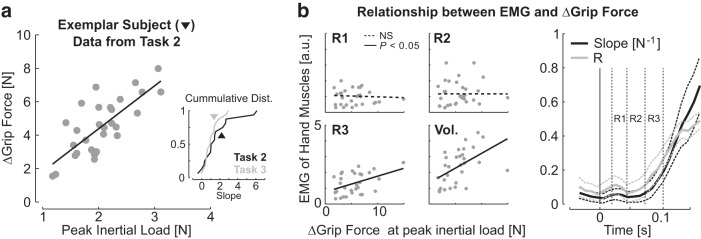
***a***, Trial-by-trial modulation of grip force with the peak inertial loads from one representative subject. The side plot presents the cumulative distributions of modulation slopes across participants in black and gray for Tasks 2 and 3, respectively. The black triangle corresponds to the exemplar participant. ***b***, Relationship between hand EMG and increments of grip force measured at the time of peak inertial load. The four panels in the left illustrate the gradually better correlation between the change in grip force and the EMG averaged across the different epochs for one representative subject. Data from individual trials were normalized to the grand-average from the pre-perturbation epoch. Right, Regression slopes and correlations between changes in grip force and the average EMG. Dashed traces represent 1 SEM.

We then addressed how the grip feedback response related to the well documented relationship between grip force modulation and inertial loads (Flanagan and Wing, 1993). These analyses were restricted to the OUT perturbations to concentrate on trial-by-trial modulation of grip force independent of the categorical changes induced by the location of the goal target. First, we did not observe any statistical relationship between the average grip force prior to the perturbation and the perturbation-related modulation across participants (mixed linear model, *p*>0.05). Thus, the analyses below concentrate on the increments of grip force measured relative to the baseline grip force (5.5 N on average during Task 2, range across participants: 2.6–8.5 N). We found that the changes in grip force measured at the peak inertial load, computed from joint kinematics (Fig. 5*a*; ΔGrip Force), were significantly correlated with the peak inertial loads for 11/16 subjects (Fig. 5*a*; linear regressions, *p*<0.05).

The focus on peak inertial loads was motivated by the fact that it is the most critical constraint that the grip force must overcome to succeed at the task, but the relationship between grip force and upper-limb feedback control started earlier. Indeed, the trial-by-trial modulation of grip force can be traced back to the onset of the hand muscle response. Paralleling previous work on trial-by-trial correlation between long-latency responses and behavioral performance (Crevecoeur et al., 2013), we quantified the link between motor responses in hand muscles and the overall grip-force modulation across epochs. A continuous estimate of the correlation and slope of the linear regression was obtained with a centered 30-ms-wide sliding average. EMG of hand muscles across epochs displayed gradually better correlation with the increments of grip force, and this relationship clearly started within the long-latency epochs (Fig. 5*b*). Recall that the peak inertial load occurred at about 260 ms on average. We performed a similar analysis based on a generalized mixed model by pooling data from all participants together and including individual differences as a random factor. This analysis revealed similar results: the overall fit started becoming significant for the bin centered on 65 ms, and the general slope rose to similar values as the average of the slopes computed across individuals.

Considering that the kinematics of corrective movements is directly linked to long-latency responses in the shoulder muscle (Crevecoeur et al., 2013), the foregoing analysis suggests that long-latency feedback in hand muscles was in fact coupled with upper-limb feedback corrections. This coupling could be observed directly. A mixed linear regression model including the participant as a random factor revealed that the relationship between EMG in hand muscles and the shoulder muscle were highly significant in the R2 and R3 epochs (*p*<0.001). When performing the regressions on individual participants’ data, we observed that hand and shoulder EMG responses were significantly correlated for 4, 5, 8, and 13/16 participants in R1, R2, R3, and Vol epochs respectively. The partial rank-correlation between hand muscles and shoulder muscles responses, obtained by controlling for the variability of the pre-perturbation activity, significantly increased across epochs (one-way ANOVA; *F*_(3,60)_=4.5, *p*<0.01). Thus, the statistical relationship between shoulder and hand muscle responses is gradually stronger across epochs.

**Figure 6.**
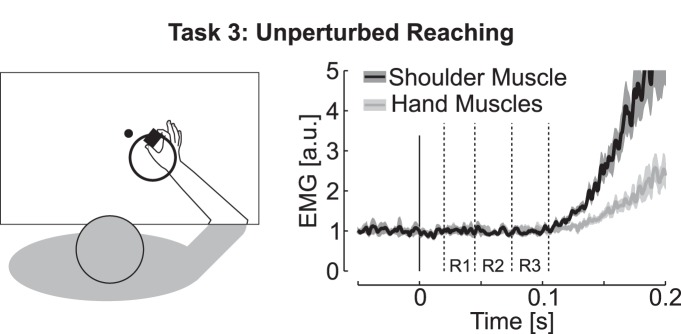
Sketch of Task 3 (left) and associated muscle response (right). Only the nearer target is represented for correspondence with muscles recordings shown on the right. However, both farther and nearer targets were used in this task in a randomized way. Dashed lines represent the different epochs as used for characterizing motor responses in Tasks 1 and 2. Observe that both shoulder and hand muscles start to increase after the end of the R3 epoch.

Finally, Task 3 was designed to measure the onset of voluntary reaction times and address possible influence of visuomotor feedback (Fig. 6). We found that EMG response onset for this task occurred later, as the activity in R3 was not significantly different from the pre-perturbation activity for both shoulder and hand muscles (R3≈Pre; *t*_(15)_<0.85, *p*>0.2), despite minimal uncertainty about the movement to perform. This result rules out rapid visuomotor pathways as contributors of the initial grip feedback response observed in Task 2. The baseline shoulder activity in this task was increased by 6% on average in comparison with the baseline in Task 2, and this increase was statistically significant (Pre from Task 3 > Pre from Task 2; *t*_(15)_=2.48, *p* <0.05). We computed linear regressions between the baseline activity and the moment when velocity exceeded 5% of its peak value and found a significant correlation for 2/16 participants. Thus, changes in baseline across trials were not correlated with reaction times or with peak velocity. Similar results were obtained when computing linear regressions between the peak velocity and the baseline activity.

We further used the data from Task 3 to compare grip force modulation during voluntary reaching with the grip force modulation observed in Task 2. We focused on movements toward the nearer target involving shoulder extension to match the movement direction across these two tasks (Fig. 6). The peak acceleration toward the target occurred later in Task 3 than in Task 2 (264±17ms in Task 2; 316±18ms in Task 3), and was smaller in absolute value (6.7±1.8 ms^−2^ in Task 2; 4.2±2.6ms^−2^ in Task 3). The later occurrence of the peak acceleration was expected as a result of the fact processing visual cues to reach for the target is typically slower than when responses are evoked by a mechanical perturbation as in Tasks 1 and 2. However, the slope of the linear regressions between grip force increments and load force peaks were statistically similar across the two tasks [median slope and interquartile range: (0.45, 1.22, 2.48) for Task 2 and (0.48, 0.94, 1.4) for Task 3; Fig. 5*a*, side plot; *t*_(15)_ = 1.72, *p*>0.1]. To summarize, grip force modulation gain was similar across unperturbed reaching and feedback control, and the relationship between increments of grip force and hand muscles could be traced back to the long-latency epoch in which upper-limb and hand motor systems exhibited synchronized and target-directed feedback.

## Discussion

A hallmark of sensorimotor systems is the ability to adjust feedback control to the environment and constraints of the task at hand (Todorov and Jordan, 2002; Scott, 2012). Here we exploited this aspect of motor control to investigate how healthy humans maintain stable holding of an object following mechanical perturbations. Two important features of grip feedback control emerged. First, the categorical differentiation of muscle responses according to the location of the goal target occurred virtually at the same time in shoulder and hand muscles (a little more than 60 ms). Second, grip feedback control rapidly expressed a tight modulation with the kinematics of upper-limb movements (Task 2), which was statistically similar to the grip-load coupling observed during voluntary reaching (Task 3). This coupling between upper-limb and grip control was observed across trials within a single condition (OUT trials), showing that the grip force modulation was not a default increase but instead shared a common source of variability in the response planning or in the generation of the motor commands. Altogether, the results highlighted that upper-limb and grip feedback responses exhibited task- and movement-dependent modulation in a synchronized way.

Dissecting the sensorimotor mechanisms underlying the grip feedback response is an important challenge for prospective studies. In particular, the earliest EMG response collected in hand muscles was undifferentiated across IN and OUT perturbation trials (Fig. 3*a*; ∼45–60 ms). The neural basis of this default response is unclear and our dataset does not allow us to investigate the details of the mechanical interactions at the finger–object interface. We extracted the end-point kinematics from shoulder and elbow angles assuming rigid bodies, but factors such as small amounts of free motion between the exoskeleton and the arm, small changes in grasp configuration, and skin compliance, likely had an impact on the transmission of the perturbation load to the object. However, we were able to measure that, on average, delays associated with such mechanical filtering of the perturbation did not exceed ∼15 ms relative to the shoulder perturbation onset (see Results). Recall that the rise time of the commanded perturbation was 10 ms, thus our data suggest fairly direct transmission of the perturbation load to the object dynamics, although the nature and timing of the perturbation at the finger–object interface in such a task remains to be characterized in more detail.

Response onset in hand muscles occurred at ∼45 ms, on average, relative to shoulder perturbation onset (Fig. 3*a*), which is compatible with short-latency response to a change in joint configuration occurring at ∼15 ms (Matthews, 1984), or with long-latency responses evoked by earlier changes in grasp configuration as observed in the upper limb following small and transient perturbations (Jaeger et al., 1982; Pruszynski et al., 2008). Cutaneous feedback is another candidate contributor to the initial response of hand muscles. Fingertip feedback is known to convey information about the fingertip–object interface that is critical for skilled manipulation (Witney et al., 2004; Johansson and Flanagan, 2009). Previous studies suggest that cutaneous afferents may engage a long-latency response (Häger-Ross et al., 1996; Macefield and Johansson, 2003), although these studies did not clearly dissociate the respective contributions of cutaneous and muscle afferent feedback. To our knowledge, quantifying how much cutaneous and muscle afferents participate to the generation of grip feedback response following perturbations remains to be thoroughly investigated.

The default EMG response observed in hand muscles was followed by a task-dependent response reflecting internal changes in the control policy, as the modulation in muscle activity preceded changes in movement kinematics or grip force. Hence, goal-directed grip feedback was generated through pathways having sufficient sophistication to incorporate target location and trial-by-trial response variability in the scaling of grip force. The latency of the goal-dependent response (∼60 ms) allows sufficient time to include the contribution of a transcortical pathway (Matthews, 1991; Pruszynski et al., 2011b). Despite the small amplitude of long-latency modulation observed in hand muscles, the behavioral relevance of this modulation was supported by the fact that it reflected goal-directed modulation (Fig. 3), and was statistically linked to the overall grip force modulation across trials (Fig. 5). Thus, although the long-latency responses in hand muscles may not be sufficient to ensure stable grasp control on their own, these responses were clearly the onset of the flexible- grip feedback response.

Our results provide important insight on the neural implementation of grip-force adjustments. During reaching, it is commonly assumed that forward models sequentially process the efferent copy of motor commands, which in turn generates anticipatory compensation for changes in load resulting from moving the held object (Kawato, 1999; Wolpert and Ghahramani, 2000; Wolpert and Flanagan, 2001). It is important to realize that, in the context of a perturbation, such an implementation predicts that the latency of the task-dependent response in hand muscles should be delayed relative to shoulder muscles. The first source of delay is internal processing time, as the generation of motor commands for hand muscles can only start after the task-dependent modulation in upper-limb motor commands. The basic concept is that motor commands for the upper limb are processed to predict the corresponding motor commands to hand muscles required to maintain grasp. This prediction is expected to take some time: for instance, the cerebro-cerebellar loop, often suggested as the route of forward models (Miall et al., 1993; Bastian, 2006; Shadmehr and Krakauer, 2008), takes 10–25 ms (Allen and Tsukahara, 1974). Other pathways projecting to interneurons in the spinal cord and back to the cerebellum have been recently shown to convey efferent copy of motor commands in rodents (Alstermark and Isa, 2012; Azim et al., 2014). It is unclear how long this loop takes in humans if similar pathways are engaged, but it is reasonable to expect delays similar if not longer than the cerebro-cerebellar feedback latency, because neural signals must travel through a longer route (from cortex to spinal cord and back up to the cerebellum).

A second source of delay that is expected to occur in the serial implementation framework is the additional time required for motor commands to travel to the more distal musculature of hand muscles in comparison with shoulder muscles. Considering motor nerve conduction velocity of 60 m/s (Ingram et al., 1987; Wang et al., 1999) and ∼70 cm of arm length on average (Winter, 1979), the difference in goal-directed EMG response latency due to conduction times should be ∼10 ms, which is compatible with the measured latency of motor-evoked potentials in shoulder and hand muscles following transcranial magnetic stimulation (Devanne et al., 2006). Thus, a sequential processing of upper-limb motor commands fed into a forward grip force controller following a perturbation predicts ≥20 ms between the expression of goal-directed motor responses at shoulder and hand muscles. Our results are not compatible with this prediction: we measured near zero difference between the latency of task-dependent response in hand and shoulder muscles (Fig. 4). Small changes in our sample, obtained by randomly removing 10% of trials, generated differences between −2.5 and 4.3 ms in 50% of cases, and the estimates in distribution were significantly shorter than 20 ms (Fig. 4*c*). In fact, the value of 20 ms corresponded to the 99th percentile of the distribution obtained from this procedure (*p* = 0.01).

The foregoing arguments suggest that a serial prediction of the consequences of motor commands does not account for the simultaneous expression of goal-directed feedback in upper-limb and hand muscles. Without rejecting the contribution of forward models in general, our results show that in Task 2, other mechanisms were responsible for the modulation of grip control following the perturbation. The data also clearly show that changes in grip force are not a default increase independent of the upper-limb feedback responses. One possible mechanism is the triggering of a pre-planned action by the perturbation (Koshland and Hasan, 2000; Shemmell et al., 2009; Shemmell, 2015). Recall however that the perturbation direction was not predictable, thus such a mechanism needs feedback about the perturbation direction. In addition, the hypothesis of a triggered response is not fully compatible with the known scaling of long-latency responses with task requirements (Pruszynski et al., 2011a), and with the fact that long-latency responses scale with unexpected changes in load magnitude provided that the temporal profile is known (Crevecoeur and Scott, 2013). These previous studies supported the idea that upper-limb motor responses resulted from a goal-directed feedback control law that takes sensory feedback as input. Importantly, these previous conclusions about long-latency responses to mechanical perturbations apply to the present experiment, because we were using the same experimental paradigm.

Thus, building on these previous results, we believe that feedback coordination provides a more parsimonious account of our data. Indeed, this framework captures well the dependency of the motor response upon the location of the goal target, and readily explains the concomitant expression of goal-directed motor response in a framework that is compatible with previous work. In this view, a form of prediction occurred during movement planning, whereby upper-limb and hand muscles are coupled together in the control law according to the location of the goal target and to the presence of a held object, but such a mechanism does not need an efferent copy of motor commands because it is pre-planned before the perturbation is applied. Clearly, the target was visible prior to the perturbation and was used to plan the response. Thus, the prepared motor response specifies control laws for both upper-limb and hand muscles to move the arm (and object) to the new spatial goal. These responses are reminiscent of the launching of a prepared action such as when a loud acoustic stimulus evokes a startle response (Carlsen et al., 2004; Honeycutt and Perreault, 2012). However, in the case of a mechanical perturbation applied to the limb, the motor system must not only launch a prepared response, but also control for the perturbation-related motion. Thus, in our experiment, the triggered response or reaction is actually a triggered control policy that must take feedback about the perturbation direction and magnitude into account.

Formally, current theories based on optimal feedback control capture this coordination of motor systems to attain a given behavioral goal (Todorov and Jordan, 2002). Indeed, this mathematical formalism expresses how one or several effectors are coupled in the control law dependent on the metric and constraints of the task, thereby unifying planning and feedback processes under a common framework. In addition, the absolute size of the motor response of shoulder muscles was also reduced likely to ensure grasp was maintained when moving to the goal. Altogether, motor responses can be rapidly scaled and appropriately coordinated to attain a complex behavioral goal, such as holding an object stable in the presence of external perturbations.

Our results do not reject the presence of forward models predicting the consequences of motor commands sequentially, which in this experiment may clearly influence later epochs of grip feedback control. It is also possible that the concomitant expression of target-directed activity in our experiment was facilitated by the fact that our task does not involve many degrees of freedom, and that more complex tasks (including more degrees of freedom) require additional processing from internal models. Clear evidence supporting the existence of forward models can be found in the visual system, in which copies of motor commands were shown to monitor the outcome of saccadic eye movements (Sommer and Wurtz, 2008). As well, state estimation requires internal processing of motor commands to maintain internal representations of the current state of the body (Vercher and Gauthier, 1992; Wolpert et al., 1995). Hence, internal predictions from forward models are clearly an important feature of motor control (Wolpert and Ghahramani, 2000; Todorov, 2004). However, we show that such predictions are not the only mechanism responsible for grip force adjustments when we manipulate objects. In particular, we highlight the presence of a more direct mechanism enabling concomitant feedback responses of upper limb and hand muscles following an external perturbation.

The relatively simple coordination mechanism was highlighted here in the context of responding to a mechanical load, but it may also be engaged more generally during voluntary control. Indeed, previous studies have suggested that voluntary control and feedback control are functionally similar in a broad range of contexts such as multi-joint coordination (Hollerbach and Flash, 1982; Kurtzer et al., 2008; Weiler et al., 2015), state estimation (Vercher and Gauthier, 1992; Crevecoeur and Scott, 2013), bimanual control (Diedrichsen, 2007; Omrani et al., 2013), and motor learning (Wagner and Smith, 2008; Cluff and Scott, 2013). The present paper further emphasizes qualitatively similar features of grip force modulation across feedback control and unperturbed reaching. Thus, in principle, there is no reason why such mechanism should not be considered as a contributor of grasp control in general and also in particular during unperturbed reaching. Clarifying how much feedback coordination or internal predictions from forward models contributes to grip-force adjustments is an important challenge for prospective studies.

## Synthesis

The decision was a result of the Reviewing Editor Trevor Drew and the peer reviewers coming together and discussing their recommendations until a consensus was reached. A fact-based synthesis statement explaining their decision and outlining what is needed to prepare a revision is listed below. The following reviewers agreed to reveal their identity: Eric Perreault, Adrian Haith

General Synthesis and Significance

The data presented in the manuscript address the important issue of how we generate rapid, coordinated responses to unexpected perturbations encountered in complex tasks. They alone will help advance the field. The findings place important constraints on potential mechanisms by which such responses are generated and coordinated. However, the questions framed in the introduction do not address plausible alternative hypotheses, and all analysis and discussion is framed in response to these questions. This greatly weakens the interpretation of an otherwise nice set of experiments.

In particular, the idea that the perturbation releases a pre-prepared action (or policy) that might have originally depended on a forward model is an important alternative interpretation that the authors should discuss.

Nevertheless, although not conclusive, the results definitely make an important contribution to the ongoing debate on forward models - a topic that is widely misconstrued as fully understood.

Reviewer #1

This paper addresses the important issue of multijoint coordination in response to postural perturbations. Specifically, the investigators examine the rapid adjustments in grip force that follow an unexpected perturbation of arm posture. They are commended for investigating such a complex task with obvious functional relevance. However, there are many methodological and interpretation issues that weaken my enthusiasm for an otherwise creative study.

A major concern with the experimental paradigm is the lack of direct endpoint measures. Angular measurements of the shoulder and elbow were used to estimate endpoint acceleration, and also as a reference for time at which the hand received sensory inputs from the perturbation. It is hard to imagine that there was not some transmission delay between the proximal limb sensors and the movements of the hand. Consequently, all discussions regarding the timing of the earliest hand muscle responses must be called into question. If the authors wish to comment on the timing of the hand EMG relative to the proprioceptive input, they will need an accelerometer or other measurement system on the held object. If they wish only to comment on the relative timing of hand and shoulder muscles, this is not needed but the Discussion will need to be changed substantially.

It is interesting that there was a decrease in the observed perturbation response when holding an object. Was a similar decrease observed in the voluntary movement trials? Was there also a corresponding difference in the reaction times or speed of movement?

The key finding is that the target-dependent perturbation responses elicited in the hand and shoulder occurred at a similar latency. This is a nice and interesting result. However, I am not at all convinced by the argument that this reflects a coordinated feedback response. Could this not simply be the rapid release of a coordinated prepared response? It would be easy to generate an appropriate forward model of the required coordination in advance, and there is a growing body of literature (completely ignored in this manuscript) suggesting that prepared actions can be elicited early by sensory stimuli including perturbations. This possibility should be discussed in detail, as it seems to be a more parsimonious explanation for the observed results.

Much space is devoted to arguments against the nervous system performing serial predictions in response to a perturbation. This is a straw man of an argument. The goal-dependent motor commands relevant to these experiments are completely predictable, even if the perturbation directions are not. There is no need for serial prediction only after the perturbation is received. As such, the experiments shed little light on the notion of plausible internal models. This does not mean that the data are not useful, just that the interpretation should be tempered.

The analysis of a correlation between hand and shoulder EMGs should be performed on the population, not individual subjects. Subjects should simply be treated as a random factor and all data fit using a generalized model. This will allow for an appropriate representation of group results, which is not available in the present manuscript. This same approach should be used for the correlation between grip force and peak inertial loads.

Could both of the above correlations not also be attributed to a simple scaling of a prepared response? The observation of similar grip force modulation in Task 3 seems to support this interpretation. If not, please explain.

Reviewer#2

In this article, the authors examine the timing of goal-directed responses to a perturbation to the arm. They compare the timing of primary responses in arm muscles to the timing of responses in hand muscles while gripping a held object. The authors argue that goal-directed changes in grip force are apparent at very short latency (∼65ms) following perturbation onset - comparable to the latency of goal-directed responses in arm muscles.

Major Concern:

A fundamental problem with the sliding window analysis is that the estimated timing of the onset of the response is strongly dependent on 1) the extent of the difference in EMG across trial types, 2) on the variability of this difference, and 3) on the chosen significance level (Figure 4b). Given that EMGs for the different effectors differ significantly wrt 1) and 2), I believe it is very difficult to justify directly comparing these estimates. Furthermore, even if one does directly compare them, the conclusion one reaches is entirely dependent on the fairly arbitrary choice of significance level - for p=0.01 one would reach a very different conclusion from p=.05. Though the authors acknowledge this difficulty, their justification for choosing p=0.05 is simply that choosing a more cautious p-value is 'problematic'. I don't believe that this is sufficient justification. This weakness undermines the very interesting core result that the grip force is modulated at a comparable latency to the arm EMG.

In order to strengthen the validity of their conclusions, I recommend the authors consider some alternative analyses that avoid this power/bias problem inherent in the present approach. One possibility would be to use the ROC criterion to estimate the response latency for the arm, then use a straightforward t-test to determine whether there is a difference in hand EMG across trial types at that latency. An alternative approach would be to fit a parametric model (e.g. a piecewise linear model) to the difference in EMG signals or the sliding-window ROC such that the response onset is a parameter of this model. It would also be very helpful to systematically determine the extent of any biases based on fits to synthetic data.

I also find the ROC analysis is not explained in sufficient detail and the motivation for using this particular approach is unclear (other than following precedent of prior papers). The authors first establish tuning of the responses based on a straightforward t-test of the mean EMG over a fixed window (R1, R2, R3, etc) across trials. It would be very helpful to better motivate the switch to an ROC analysis for the sliding window. Could one not simply perform a t-test on the EMG data in a sliding window? What is the advantage of computing an intermediate ROC measure (particularly given that this significantly reduces the transparency of the analysis)? The fact that a direct comparison of the EMG is a reasonable thing to do is clear from line 223, where the authors appeal to the (near-)significant difference in EMG in R2 to help justify the threshold they select for the significance for the ROC sliding window analysis. If that is the strength of the argument for choosing p=0.05, then why not simply skip the ROC and just compare the EMGs with a t-test in a sliding window?

Minor Concerns:

Opening line of the abstract: "Suppose that someone bumps into your arm at a cocktail party while you are holding a glass of wine." - Presumably, however, one would be holding a cocktail at a cocktail party, rather than a glass of wine.

Figure 5: why does this analysis examine changes in grip force and not absolute grip force? One might expect that subjects who had a strong baseline grip wouldn't need to modulate their grip force so much to satisfy a safety margin. It would be nice to at least comment on the distribution of baseline grip forces.

What time did the peak inertial load typically occur? This would be helpful to know in interpreting e.g. Fig 5b.

The right panel of Fig 5b is not explained in sufficient detail. What size window of data was used for the correlation?
